# Years of life lost due to infectious diseases in Poland

**DOI:** 10.1371/journal.pone.0174391

**Published:** 2017-03-23

**Authors:** Marek Bryla, Elzbieta Dziankowska-Zaborszczyk, Pawel Bryla, Malgorzata Pikala, Irena Maniecka-Bryla

**Affiliations:** 1 Department of Social Medicine, the Chair of Social and Preventive Medicine of the Medical University of Lodz, Lodz, Poland; 2 Department of Epidemiology and Biostatistics, the Chair of Social and Preventive Medicine of the Medical University of Lodz, Lodz, Poland; 3 Department of International Marketing and Retailing, University of Lodz, Lodz, Poland; TNO, NETHERLANDS

## Abstract

**Purpose:**

An evaluation of mortality due to infectious diseases in Poland in 1999–2012 and an analysis of standard expected years of life lost due to the above diseases.

**Methods:**

The study material included a database created on the basis of 5,219,205 death certificates of Polish inhabitants, gathered between 1999 and 2012 and provided by the Central Statistical Office. Crude Death Rates (CDR), Standardized Death Rates (SDR) and Standard Expected Years of Life Lost (SEYLL) due to infectious and parasitic diseases were also evaluated in the study period as well as Standard Expected Years of Life Lost per living person (SEYLL_p_) and Standard Expected Years of Life Lost per dead person (SEYLL_d_). Time trends were evaluated with the application of joinpoint models and an annual percentage change in their values.

**Results:**

Death certificates report that 38,261 people died due to infectious diseases in Poland in the period 1999–2012, which made up 0.73% of the total number of deaths. SDR caused by these diseases decreased, particularly in the male group: Annual Percentage Change (APC = -1.05; 95% CI:-2.0 to -0.2; p<0.05). The most positive trends were observed in mortality caused by tuberculosis (A15-A19) (APC = -5.40; 95% CI:-6.3 to -4.5; p<0.05) and also meningitis, encephalitis, myelitis and encephalomyelitis (G03-G04) (APC = -3.42; 95% CI:-4.7 to -2.1; p<0.05). The most negative mortality trends were observed for intestinal infectious diseases (A00-A09) Annual Average Percentage Change (AAPC = 7.3; 95% CI:3.1 to 11.7; p<0.05). SDR substantially decreased in the first half of the study period, but then significantly increased in the second half. Infectious and parasitic diseases contributed to a loss of around 37,000 standard expected years of life in 1999 and more than 28,000 in 2012. During the study period, the SEYLL_p_ index decreased from 9.59 to 7.39 per 10,000 population and the SEYLL_d_ index decreased from 14.26 to 10.34 years (AAPC = 2.3; 95% CI:-2,9 to -1.7; p<0.05).

**Conclusions:**

Despite smaller numbers of deaths reported from infectious causes these diseases still represent a serious problem for Poland compared to countries in Western Europe.

## Introduction

The ongoing changes in global health, known as epidemiological transition, have given rise to a different model of mortality characterised by a declining share of infectious diseases and a growing share of non-communicable diseases [1]. However, infectious diseases still pose a serious problem for public health, particularly in developing countries, where they are the most common factors contributing to mortality and disability. Globally, they constitute the second most common cause of death [2]. In contrast, due to the development of effective prevention and treatment methods of infectious diseases in developed countries, non-communicable diseases are a more common cause of mortality in those countries. However, these diseases still represent an important problem by contributing to a huge number of years of life lost, as confirmed by the SEYLL_d_ index. Well-known infectious and parasitic diseases which can be easily countered by current medical methods are being replaced by new ones and modern doctors often lack the knowledge to cope with them. This problem is further exacerbated by the growing phenomenon of antibiotic resistance. Infectious diseases are often a cause of complications in non-communicable diseases and frequently lead to death. They also play a key role in the formation of certain neoplasms, such as hepatitis C, hepatocellular carcinoma, HPV and cervical cancer.

In response to the threat of bioterrorism using genetically-modified pathogens, and to allow social mobility in the modern world to be studied, the WHO, the European Commission and the European Centre for Disease Prevention and Control (ECDC) recommend monitoring the epidemiological situation regarding infectious diseases in particular countries [3].

DALY (Disability-Adjusted Life Years) is a measure which comprises years of life lost (YLLs) and years of life with disability (YLDs). It reflects the falling mortality associated with the group of infectious diseases over time at the expense of non-infectious diseases, which is mostly caused by the growth and ageing of the world population and the improvement of its socio-economic conditions. Among all the infectious diseases analyzed by *Global Burden of Disease Study*, the standardized DALY measure was found to increase only for Dengue (from 16.6 to 25.5 per 100,000) and for Ebola (from 0.0 to 3.9 per 100,000) in the period 2005 to 2015. In 2015, while lower respiratory infections, diarrheal diseases and HIV/AIDS were the third, sixth and tenth highest causes of DALY globally, no infectious disease was found among the ten main causes of DALY in Poland or across the rest of Europe [4].

The share of infectious diseases in the mortality model in Poland has declined significantly, amounting to 23.7% of all deaths in 1925 [5], but only 0.5% in 2014 [6]. In the period under study, infectious diseases caused 2476 deaths in Poland in 1999, which constituted 0.65% of all deaths, and 0.68% of all deaths in 2012. As the mortality risk is high for certain diseases, including untreated HIV and AIDS and meningitis, this results in life years lost due to premature mortality caused by infectious diseases.

The aim of the study is to evaluate changes in mortality due to infectious diseases in the period 1999–2012 in Poland, according to SEYLL, SEYLL_p_ and SEYLL_d_.

## Methods

Our database includes the underlying causes coded according to the International Statistical Classification of Diseases and Health Related Problems—Tenth Revision (ICD-10). According to the ICD the following diseases are considered infectious and parasitic [7]:

- certain infectious and parasitic diseases (A00-B99), including:
- intestinal infectious diseases (A00-A09),
- tuberculosis (A15-A19),
- certain zoonotic bacterial diseases (A20-A28)
- other bacterial diseases (A30-A49),
- infections with a predominantly sexual mode of transmission(A50-A64),
- other spirochetal diseases (A65-A69),
- other diseases caused by chlamydia (A70-A74),
- viral infections of the central nervous system (A80-A89),
- viral infections characterized by skin and mucous membrane lesions (B00-B09),
- viral hepatitis (B15-B19),
- human immunodeficiency virus [HIV] disease (B20-B24),
- other viral diseases (B25-B34),
- mycoses (B35-B49),
- protozoal diseases (B50-B64),
- helminthiases (B65-B83),
- sequelae of infectious and parasitic diseases (B90-B94),
- bacterial meningitis, not elsewhere classified (G00),
- meningitis due to other and unspecified causes, encephalitis, myelitis and encephalomyelitis (G03-G04),
- salpingitis and oophoritis, inflammatory disease of the uterus, except the cervix, inflammatory disease of cervix uteri, other female pelvic inflammatory diseases (N70-N73).

It is worth noting, however, that standard reporting methods do not record the specific infectious agent that contributed to the death.

A database was formed from a total of 5,219,205 death certificates, which were issued in Poland during the period 1999–2012. This information was obtained from the Central Statistical Office. An initial inquiry into the database found that 38,216 of the deaths were caused by infectious or parasitic diseases (S1 File). As parasitic diseases are uncommon in Poland, the term: ‘infectious diseases’ was used for the analysis.

Mortality due to infectious diseases was calculated based on the CDR and SDR. Standardization was carried out by a direct method, with the population of Europe assumed as the standard.

The SEYLL index was used to calculate the morbidity attributable to infectious diseases [8–9]. The SEYLL index is used to calculate the number of years of life lost by the studied population due to a certain cause in comparison with the years lost by the standard population. Standard expected years of life were calculated based on the *Coale and Demeny West Level 26* model life tables [10], corresponding to the expected years of life for Japanese residents, according to which the number of standard expected years of life was 82.5 for a woman and 80 for a man.

Standard expected years of life lost (base version) were calculated with the use of the following formula:

$$SEYLL=\sum_{x=0}^{l}d_{x}e_{x}$$

where, d_x_ stands for the number of deaths at age x, e_x_ is the number of expected years of life that remain to be lived by the population which is at age x, and *l* is the age of the oldest dead person.

After application of the following formula and dividing the number of lost years of life by the number of deaths the authors obtained the number of years of life lost by one dead person.

$$SEYLL_{d}=\frac{\sum_{x=0}^{l}d_{x}e_{x}}{\sum_{x=0}^{l}d_{x}}$$

By dividing the number of lost years of life by the size of the studied population (N) we obtain the number of years of life lost due to death per one person [11]:

$$SEYLL_{p}=\frac{\sum_{x=0}^{l}d_{x}e_{x}}{N}$$

In the above study the obtained value related to a population of 10,000. The standard expected years of life lost were calculated using time-discounted rates with age-weighted measures according to the following formula [9].

$$SEYLL\left[r;K\right]=\sum_{x=0}^{l}d_{x}\left\{\left(\frac{KCe^{rx}}{{\left(\beta +r\right)}^{2}}\right)\left[e^{-\left(\beta +r\right)\left(L+x\right)}\left[-\left(\beta +r\right)\left(L+x\right)-1\right]-e^{-\left(\beta +r\right)x}\left[-\left(\beta +r\right)x-1\right]\right]+\left[\frac{1-K}{r}\right]\left(1-e^{-rL}\right)\right\}$$

where the following stand for:

r—discount rate (r = 0 or r = 0.03)

K—factor modelling age valuation (K = 0 or K = 1)

d_x_—number of people who died at age x

l—age of the oldest dead person

x—age at which the person died

L—expected years of life for age x

C—constant (0.1658)

e—constant (≈2.718)

β—parameter of the function of age valuation (0.04).

A number of publications concerning deaths due to infectious diseases weight the number of years of life lost by age and discount the value by time [12–13]. As more extensive comparisons are used in the present study, it uses values calculated with the application of the methods applied in the bibliography cited above. The SEYLL presented in further parts of this study will refer to the number of years of life lost weighted by age and discounted by time, i.e. SEYLL [0.03;1].

Time trends of the analysed measures and parameters were evaluated with the use of the joinpoint method and Joinpoint Regression programme [14]. This method is an advanced version of linear regression, in which a time trend is expressed with a broken line, which is sequence of segments joined into joinpoints, Within these points, the change of the value is statistically significant (p<0,05).

The data in the present study was analysed using a linear regression model where the natural logarithm of the studied measures was a dependent variable and the calendar year was an independent variable; (x) (y = a+bx, where y = ln of the analysed measures, x = calendar year). Annual Percentage Change of the rate values for each trend was calculated according to the following formula:

$$APC=100({\text{exp}}^{b}-1)$$

In order to calculate the statistical significance of APC, its corresponding 95% confidence intervals (CI) were determined. APC was also calculated for each segment of broken lines, and Average Annual Percentage Change (AAPC) for a full range of analyzed years with corresponding 95% CI. [15]. The Monte Carlo Permutation method was used [16]. It is worth noting that in our study, negative APC values indicate a positive trend, while positive APC values mean a deterioration of the situation, i.e. an increase of SDR and SEYLL measures in the period under study.

The Bioethics Committee of the Medical University of Lodz gave consent for the study to be conducted (No. RNM/422/12KB of 22 May, 2012).

## Results

Infectious diseases (according to the ICD-10: A00-B99, G00, G03-G04, N70-N73) contributed to 0.73% of deaths in Poland in the study period. In the period 1999–2012, the values of both CDR and SDR due to infectious diseases fluctuated by year. The values of real and standardized death rates due to infectious diseases were higher in the male group.

In 1999, the CDR value associated with infectious diseases for males was 8.95 (Table 1). The rate reached its maximum value in 2011 (10.24 per 100,000 males) and its minimum value in 2012 (8.24 per 100,000 males). The rate slightly grew by a mean value of 0.41% per year during the period 1999–2012 (p>0.05). In females, the values were 4.62 per 100,000 females in 1999 and 6.12 per 100,000 females in 2012. A significant mean annual growth rate of 2.89% was observed in the female group (p<0.05).

**Table 1:** CDR and SDR (per 100,000 population) due to infectious diseases in Poland in 1999–2012 by sex.

Years	CDR			SDR		
	Men	Women	Total	Men	Women	Total
1999	8.95	4.62	6.72	10.27	4.35	6.96
2000	9.32	5.01	7.10	10.54	4.52	7.19
2001	8.70	4.74	6.66	9.66	4.26	6.64
2002	8.83	4.99	6.85	9.78	4.37	6.76
2003	9.21	4.59	6.82	10.05	3.81	6.52
2004	8.54	4.52	6.47	9.19	3.75	6.12
2005	8.82	4.63	6.66	9.27	3.78	6.20
2006	8.76	4.97	6.81	9.12	4.01	6.28
2007	8.49	4.81	6.59	8.62	3.81	5.96
2008	9.72	5.84	7.71	9.70	4.50	6.82
2009	9.77	6.27	7.96	9.71	4.69	6.90
2010	9.69	6.13	7.85	9.46	4.27	6.60
2011	10.24	7.15	8.64	9.88	4.96	7.14
2012	8.24	6.12	7.14	7.76	4.05	5.75

CDR = crude death rate; SDR = standardized death rate

**Fig 1:**
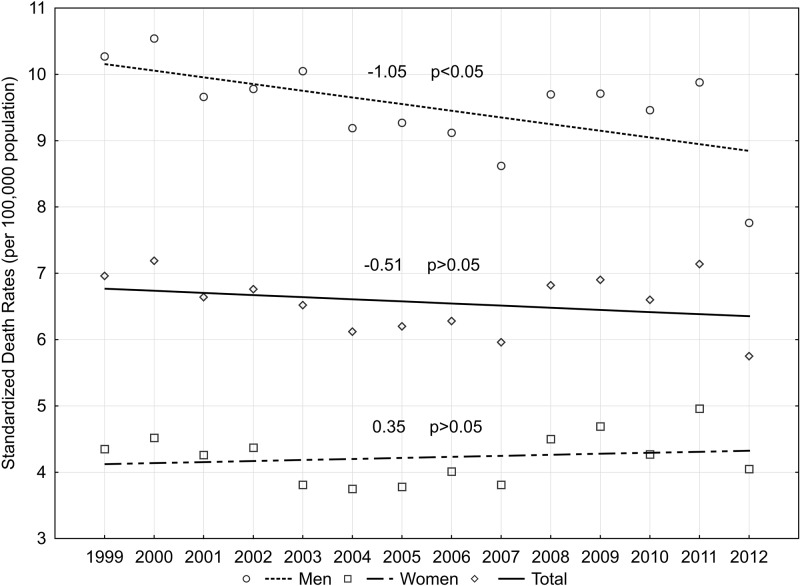
SDR trends due to infectious diseases (per 100,000 population) and annual percentage change, for the total population of Poland and by sex in 1999–2012.

In the population of Poland as a whole, CDR values due to infectious diseases increased significantly by 1.37% per year in the period 1999–2012 (p>0.05). Negative changes in the values of CDR due to infectious diseases were seen as a result of the process of ageing of Polish society; as indicated by the SDR values during the period. From 1999 to 2012, the values changed from 10.27 to 7.76 per 100,000 males, 4.35 to 4.05 per 100,000 females and 6.96 to 5.75 per 100,000 total population. Across the whole study period, SDR values due to infectious diseases decreased in the male population (APC = -1.05%; 95% CI:-2.0 to -0.2; p<0.05), increased in the female population (APC = 0.35%; p>0.05) and slightly decreased for the total population of Poland (APC = -0.51%; p>0.05) (Fig 1).

In the period under study, 90–95% of deaths due to infectious diseases were caused by intestinal infectious diseases (A00-A09), tuberculosis (A15-A19), other bacterial diseases (A30-A49), viral hepatitis (B15-B19), human immunodeficiency virus [HIV] disease (B20-B24), bacterial meningitis, not elsewhere classified (G00), meningitis due to other and unspecified causes, encephalitis, myelitis or encephalomyelitis.

A decrease in SDR due to tuberculosis (APC = -5.49; 95% CI:-6.3 to -4.6; p<0.05 for males; APC = -6.00; 95% CI:-7.5 to -4.4; p<0.05 for females and APC = -5.40; 95% CI:-6.3 to -4.5; p<0.05 for the whole Polish population) positively affected the change in the SDR value due to the analysed diseases in the population of Poland in the study period (Table 2). Also, the SDR values due to meningitis, encephalitis, myelitis and encephalomyelitis (G03-G04) decreased systematically in this period (APC = -2.71; 95% CI:-4.9 to -0.5; p<0.05 for males; APC = -4.27; 95% CI:-6.0 to -2.5; p<0.05 for females and APC = -3.42; 95% CI:-4,7 to -2.1; p<0.05 for the whole Polish population). It should be stressed that in the male group, SDR values due to bacterial meningitis (G00) decreased between 1999 and 2001 (APC = -21.74; p>0.05) but slightly increased after 2001 (APC = 0.18; p>0.05). In the female group, SDR decreased in the whole period (APC = -2.18; p>0.05). Taking both sexes together, similarly to the male group, SDR due to G00 decreased in the period 1999–2001 and slightly increased after 2001.

**Table 2:** Changes in SDR trends due to the selected infectious diseases in Poland in 1999–2012 by sex.

Causes of death		Number of joinpoints	Period	APC 95% CI	AAPC 95%CI
Infectious and parasitic diseases (A00-B99, G00, G03-G04, N70-N73)	Total	0	1999–2012	-0.51 (-1.5; 0.5)	
	Men	0	1999–2012	-1.05* (-2.0; -0.2)	
	Women	0	1999–2012	0.35 (-0.9; 1.7)	
Intestinal infectious diseases (A00-A09)	Total	1	1999–2006	-8.97* (-14.1; -3.5)	7.3* (3.1; 11.7)
			2006–2012	30.11* (20.9; 40.1)	
	Men	1	1999–2006	-11.69* (-19.0; -3.7)	6.7* (0.5; 13.2)
			2006–2012	32.92* (19.1; 48.4)	
	Women	1	1999–2005	-9.90* (-17.8; -1.2)	7.4* (2.2; 12.9)
			2005–2012	24.87* (16.1; 34.3)	
Tuberculosis (A15-A19)	Total	0	1999–2012	-5.40* (-6.3; -4.5)	
	Men	0	1999–2012	-5.49* (-6.3; -4.6)	
	Women	0	1999–2012	-6.00* (-7.5; -4.4)	
Other bacterial diseases (A30-A49)	Total	0	1999–2012	3.63* (1.6; 5.7)	
	Men	0	1999–2012	4.46* (2.0; 7.0)	
	Women	0	1999–2012	-2.98* (-1.1; -4.9)	
Viral hepatitis (B15-B19)	Total	1	1999–2010	-1.74* (-2.7; -0.0)	0.6 (-1.5; 2.8)
			2010–2012	10.31 (-5.4; 28.6)	
	Men	0	1999–2012	-1.74*(-2.7; -0.7)	
	Women	0	1999–2012	1.53 (-0.4; 3.5)	
Human immunodeficiency virus [HIV] disease (B20-24)	Total	0	1999–2012	0.37 (-1.2; 1.9)	
	Men	0	1999–2012	0.27 (-1.7; 2.3)	
	Women	0	1999–2012	0.65 (-1.9; 3.3)	
Bacterial meningitis, not elsewhere classified (G00)	Total	1	1999–2001	-21.67 (-41.1; 4.2)	-3.6 (-7.4; 0.4)
			2001–2012	0.14 (-1.8; 2.1)	
	Men	1	1999–2001	-21.74 (-43.9; 9.3)	-3.6 (-8.0; 1.1)
			2001–2012	0.18 (-2.1; 2.5)	
	Women	0	1999–2012	-2.18 (-4.8; 0.5)	
Meningitis due to other and unspecified causes, encephalitis, myelitis and encephalomyelitis (G03-G04)	Total	0	1999–2012	-3.42* (-4.7; -2.1)	
	Men	0	1999–2012	-2.71* (-4.9; -0.5)	
	Women	0	1999–2012	-4.27* (-6.0; -2.5)	

(* p<0.05)

With regards to other bacterial diseases (A30-A49), a statistically significant increase in SDR was observed in the male group (APC = 4.46; 95% CI:2.0 to 7.0; p<0.05) and a statistically significant decrease in the female group (APC = -2.98; 95% CI:-1.1 to -4.9; p<0.05). For the whole population of Poland, SDR values due to the above diseases significantly increased during the analysed period (APC = 3.63; 95% CI:1.6 to 5.7; p<0.05). With regards to viral hepatitis (B15-B19), SDR significantly decreased in the male group from 1999 to 2012 (APC = -1.74; 95% CI:-2.7 to -0.7; p<0.05) but increased in the female group over the same period (APC = 1.53; p>0.05). For the population as a whole, SDR values due to viral hepatitis significantly decreased from 1999 to 2010 (APC = -1.74; 95% CI:-2.7 to 0.0; p<0.05), but grew after 2010 (APC = 10.31; p>0.05).

SDR due to HIV (B20-B24) slightly increased over the studied period for males (APC = 0.27; 95% CI:-1.7 to 2.3) and females (APC = 0.65; 95% CI:-1.9 to 3.3) as well as for the whole population of Poland (APC = 0.37; 95% CI:-1.2 to 1.9).

It should be pointed out that with regards to intestinal infectious diseases (A00-A09), SDR values significantly decreased in the male group in the period 1999 to 2006 (APC = -11.69; 95% CI:-19.0 to -3.7; p<0.05), and this decrease was followed by a rapid and significant growth until 2012 (APC = 32.92; 95% CI:19.1 to 48.4; p<0.05). A similar observation was made in the female group: SDR significantly decreased from 1999 to 2005 (APC = -9.90; 95% CI:-17.8 to -1.2; p<0.05) and significantly increased from 2005 to 2012 (APC = 24.87; 95% CI: 16.1 to 34.3; p<0.05). Similarly, with regard to the Polish population as a whole, the study period can be divided into two parts: the period 1999 to 2006, characterized by a significant decrease (APC = -8.97; 95% CI:-14.1 to -3.5; p<0.05), and the period 2006 to 2012, characterized by a significant increase (APC = 30.11; 95% CI: 20.9 to 40.1; p<0.05).

Each premature death contributes to a loss of years of life. The number of standard expected years of life lost due to infectious diseases, calculated for the size of the population, as well as people who died in 1999–2012 fluctuated according to the year.

It is important to stress that all the calculated indices for standard expected years of life lost were usually the highest at the beginning of the analysed period and the lowest at the end (Table 3). It is also important that throughout the whole study period, the SEYLL, SEYLL_p_, and SEYLL_d_ indices (discounted by time and weighted by age) were higher in males than in females.

**Table 3:** Years of life lost due to infectious diseases in Poland in 1999–2012 by sex (values discounted by time and weighted by age).

Years	SEYLL			SEYLL_p_*10,000			SEYLL_d_		
	Men	Women	Total	Men	Women	Total	Men	Women	Total
1999	24462.40	12406.48	36868.87	13.10	6.27	9.59	14.63	13.57	14.26
2000	24581.78	12144.52	36726.30	13.26	6.16	9.60	14.22	12.30	13.52
2001	22689.56	11464.44	34154.00	12.24	5.81	8.93	14.08	12.27	13.41
2002	22768.47	11606.11	34374.57	12.30	5.89	8.99	13.93	11.78	13.13
2003	23377.02	10362.08	33739.10	12.64	5.26	8.83	13.73	11.46	12.94
2004	20863.79	10002.86	30866.65	11.29	5.08	8.08	13.22	11.23	12.50
2005	21903.22	10103.94	32007.17	11.86	5.13	8.39	13.45	11.08	12.60
2006	22076.48	10990.47	33066.95	11.97	5.58	8.67	13.66	11.21	12.74
2007	20818.92	10088.83	30907.76	11.30	5.12	8.11	13.32	10.64	12.31
2008	23560.18	12377.64	35937.82	12.80	6.28	9.43	13.17	10.75	12.22
2009	22668.81	12763.22	35432.03	12.31	6.47	9.29	12.59	10.32	11.67
2010	22025.16	10604.16	32629.32	11.88	5.35	8.51	12.26	8.73	10.84
2011	22758.63	12596.20	35354.83	12.20	6.34	9.17	11.92	8.86	10.61
2012	18440.00	10022.20	28462.20	9.89	5.04	7.39	12.01	8.24	10.34

SEYLL—standard expected years of life lost; SEYLLp*10,000 = standard expected years of life lost per 10,000 living persons; SEYLLd = standard expected years of life lost per one dead person

It should be emphasized that the number of analyzed standard expected years of life lost due to infectious diseases fell over the study period (Table 4). The authors noted a positive, significant decrease in the value of the SEYLL in males (APC = -1.04; 95% CI: -1.9 to -0.2; p<0.05). With regards to females the decrease was not statistically significant (APC = -0.31; p>0.05). Similarly, the decrease in the SEYLL_p_ index was statistically significant in males (APC = -1.04; 95% CI: -1.9 to -0.1; p<0.05) but not significant in females. It should be pointed out that similarly to SEYLL and SEYLL^p^_p_, the value of the SEYLL_d_ index (discounted by time and weighted by age) significantly decreased over the study period, which was a positive trend (AAPC = -1.6; 95% CI: -2.5 to -0.7; p<0.05 in the male group; AAPC = -3.4; 95% CI: -4.5 to -2.3; p<0.05 in the female group and AAPC = -2.3; 95% CI: -2.9 to -1.7; p<0.05 for the whole population of Poland). Information in the table confirms that while this was a decreasing trend, it fluctuated during different sub-periods.

**Table 4:** Changes in the SEYLL, SEYLL_p_ and SEYLL_d_ indices due to infectious diseases in Poland in 1999–2012 by sex.

Years of life lost		Number of joinpoints	Period	APC 95% CI	AAPC 95%CI
*Values discounted by time and weighted by age*					
SEYLL	Total	0	1999–2012	-0.79 (-1.8; 0.2)	
	Men	0	1999–2012	-1.04* (-1.9; -0.2)	
	Women	0	1999–2012	-0.31(-1.7; 1.1)	
SEYLL_p_*10,000	Total	0	1999–2012	-0.81 (-1.8; 0.2)	
	Men	0	1999–2012	-1.04*(-1.9; -0.1)	
	Women	0	1999–2012	-0.35 (-1.7; 1.0)	
SEYLL_d_	Total	1	1999–2008	-1.52* (-2.0; -1.0)	-2.3* (-2.9; -1.7)
			2008–2012	-4.12* (-5.9; -2.3)	
	Men	2	1999–2004	-1.65* (-2.6; -0.7)	-1.6* (-2.5; -0.7)
			2004–2007	-0.02 (-4.3; 4.4)	
			2007–2012	-2.57* (-3.5; -1.6)	
	Women	1	1999–2008	-2.21* (-3.2; -1.2)	-3.4* (-4.5; -2.3)
			2008–2012	-6.02* (-9.3; -2.6)	

* p<0.05; CI = Confidence interval; APC = Annual percentage change; AAPC = average annual percentage change

## Discussion

### Selection of method used to assess life years lost

Health indicators which measure premature mortality in units of time lost are gaining prominence in the assessment of the health situation of a population. This is particularly relevant for diseases with low mortality rates and which have a small share of total deaths in a given population. Infectious diseases in developed countries are an example of such diseases. In order to assess their influence on health, social, and economic loss, it is necessary to analyse life years lost due to premature mortality. The method for calculating life years lost depends on the researcher, and the choice of approach may cause problems with comparability among populations, especially on the international scale.

Various approaches to calculating these measures exist, depending on the assumed life limit. For example, the PYLL (Potential Years of Life Lost) indicator arbitrarily assumes a life limit between 60 and 85 years. The selection of this limit is often based on questionable arguments, which undoubtedly constitutes its weakness. Another disadvantage is that it ignores the benefits stemming from health interventions oriented at the oldest groups of the society.

To eliminate the weaknesses of the PYLL indicator, the PEYLL (Period Expected Years of Life Lost) indicator assumes a local period of life expectance at each age as the life limit. By doing this, it does not have an arbitrarily assumed age above which deaths are not counted to calculate the burden. However, one weakness of PEYLL is its lack of ability to make comparisons over time or across populations with various life expectancies.

This problem is eliminated in the SEYLL (Standard Expected Years of Life Lost) indicator, which assumes life expectancy based on an ideal standard to calculate years of life lost.

When calculating SEYLL values, two factors to be considered are: ‘weighting’ by age and ‘discounting’ by time. The ‘weight’ of a year of life for a person in the youngest and oldest age groups is assumed to be lower than that of a year in the life of a young person. Such an approach has been adopted by a number of authors. Murray and Lopez, for example, describe the social preferences regarding the importance of a year of life at different periods of life: i.e. children, young people and the elderly. Discounting SEYLL enables its values to be compared over time. It is assumed that effects gained at the moment are relatively more valuable than those expected in future.

### The significance of infectious diseases in the health situation of Polish residents

Between 1950 and 2000 in Poland, the majority of deaths were caused by non-communicable diseases, particularly diseases of the circulatory system and malignant neoplasms [17–18] and external causes, such as road traffic accidents and suicides [19]. In the study period, infectious diseases contributed to 0.73% of the total number of deaths. It is worth noting that the significance of infectious diseases in Poland was much higher over half a century ago, causing 6.86% of deaths in 1960. Effective prophylaxis, especially widespread vaccination and the introduction of effective methods of treatment, such as antibiotic therapy, have greatly reduced the occurrence of infectious diseases, resulting in their share in mortality falling to less than 1% since the 1990s. The improvement in the health situation of the Polish population, related to the ongoing socio-economic transition, is countered by its ageing as a result of the demographic transition. Our findings suggest that the gradual ageing of the Polish population increased the CDR value due to the presence of many diseases, including infectious diseases. Previous studies note that the subpopulation of elderly people is more susceptible to infections and responds to treatment more poorly due to their resistance mechanisms being weaker [20]. Perhaps additional reasons are that they do not see medical attention early enough and have limited access to care. In addition, the social and economic transformations occurring in Poland since 1989 have greatly improved various aspects of living conditions, resulting in improved sanitation and hygiene conditions, well-balanced diets, and healthy lifestyles. This, in turn, has decreased SDR values due to the influence of a number of infectious diseases [21]. In addition, greater infections and deaths due to AIDS have been observed in Poland caused by the global HIV pandemic.

The WHO considers avoidable mortality an important element of health policy [22]. According to the theoretical concept of avoidable mortality, premature mortality can be divided into avoidable mortality and unavoidable mortality. Deaths can be avoided by prophylaxis (preventable mortality) and by treatment and medical intervention (amenable/treatable mortality) [23–24]. It should be emphasized that this concept has many methodological limitations, such as the large number of criteria used for selecting diseases for analysis, the varying requirements concerning local conditions, and the wide range of software programs used to obtain data. The range of activities employed to fight infectious diseases may differ across countries, which also impedes comparisons [25].

### Tuberculosis mortality

In 1990 tuberculosis contributed to 1,471,500 deaths, comprising 3.16% of the total number of deaths in the world. In 2010, tuberculosis killed 1,196,000 people (2.27% of the total number of deaths). Global SDR due to tuberculosis decreased from 33.3 per 100,000 population in 1990 [26] to 18.5 per 100,000 population in 2013 [27]. A study based on the ICD-9 during the period 1965–1999/2000 confirmed that avoidable deaths caused by tuberculosis in Russia clearly outnumbered deaths caused by other infectious diseases. In Russia and the three Baltic states, Latvia, Lithuania and Estonia, deaths due to TB constituted approximately 75% of the total number of deaths due to infectious diseases [28]. In comparison, a study carried out in Greece with the use of ICD-9 covering the period 1980–2007 found a decrease in the number of deaths caused by infectious diseases (tuberculosis, pneumonia, measles, pertussis and influenza) during this time. The authors suggest that this trend might result from a better health care system, widespread use of antibiotics and vaccination, or might be caused by improved living standards resulting from epidemiological transformation [29]. Another study of a detailed analysis of 19 avoidable deaths in the period 1989 to 1997 in 17 European countries found the epidemiological situation concerning tuberculosis to improve over the period: the death rate in people aged 0–64 years old decreased from 0.68 to 0.40 per 100,000 population [30]. A comprehensive report on the epidemiological situation regarding tuberculosis published in 2015 found the SDR to be 4.1 per 100,000 population in 53 countries of the WHO European Region [31].

In Poland during the study period, positive, declining trends of mortality due to TB were observed. In 1999, tuberculosis contributed to 47.67% of the total number of deaths due to infectious diseases, and in 2012 the percentage was 22.64. In 2012, 630 people died of TB in Poland: a mortality rate of 1.6 per 100,000 population or only 0.2% of all deaths. The number of life years lost due to TB mortality amounted to 7259.45, SEYLL_p_ = 1.88, but SEYLL_d_ was 11.47 years, which means that each male who died of TB lost 12.06 years, and each female who died lost 9.56 years [32].

### HIV/AIDS mortality

During the studied period, the most significant changes in lost life years among infectious diseases on the global scale were noted for HIV/AIDS. While infectious diseases occupied twenty-ninth position in 1990, this rose to fourth position by 2005. Thanks to the improvement of the treatment of HIV/AIDS, this dropped to seventh position in 2015 [33]. In Poland, SDR due to HIV/AIDS was observed to grow at a rate of 0.37% per year throughout the entire study period, resulting in an increase in the number of life years lost due to premature mortality. According to data provided by the Institute for Health Metrics Evaluation in the USA, HIV/AIDS was responsible for 5501.79 of life years lost (0.06% of all YLL) in 1990, which grew to 8068.33 life years lost in 2005(0.11% of all YLL). [34].

### Mortality due to other infectious diseases

The remaining groups of diseases analysed in our study were characterised by declining mortality trends, with the exception of other bacterial diseases (A30-49), viral hepatitis (B15-B19) in the period 2010–2012 and intestinal infectious diseases (A00-A09) in the period 2006–2012. A decrease was observed in the number of deaths caused by viral hepatitis (B15-B19) over the whole period: 8.5% of deaths in 1999 rising to 10.4% in 2012. The increasing trend from 2010 to 2012 was probably caused by hepatitis C, as it has grown in significance in Poland, while hepatitis B has declined [35]. The increasing trends of SDR due to other bacterial diseases (A30-A49) may be related to an increase of sepsis diagnoses (A40-A41); these are a result of other diseases, for instance of the respiratory system, and sepsis should not be indicated as the underlying cause. The increasing trend of SDR due to intestinal infectious diseases observed in the period 2006–2012, following a decline in the period 1999–2006 may be due to the seasonal character of these diseases as well as the improvement in the quality of statistical data.

A report of the Central Sanitary Inspectorate in Poland considered the epidemiological situation due to infectious diseases in Poland in 2012 to be generally good. No epidemic incidence of infectious diseases was observed, although seasonal increases in the incidence of certain infectious diseases were noted, as before [36].

### Standard expected years of life lost measures (SEYLL, SEYLL_p_, SEYLL_d_)

As mentioned above, the calculation of standard expected years of life lost (SEYLL) is growing in importance as it plays a role in the evaluation of not only health but also social and economic loss [37–40]. As noted in one of our previous articles, the commonly used SEYLL_d_ enables the severity of particular diseases to be determined [41]. According to the results of the SEYLL_d_, infectious diseases are in 10^th^ position of the most serious diseases, i.e. those which most affect the health of the Polish population. The SEYLL_d_ values for infectious diseases are 12.0 for males and 8.2 years for females, indicating that the Polish population faces a serious health problem. To put the figures into context, the number of standard years of life lost for one death due to cardiovascular diseases is 5.6 years for males and 3.7 years for females, the number lost due to malignant neoplasm of the pancreas is 7.5 for males and 6.2 for females, and the number lost due to road traffic accidents is as many as 20.2 years for males and 17.1 for females.

The WHO have used the SEYLL_p_ and SEYLL_d_ indices to place Poland within a group of 39 European countries. The SEYLL_p_ and SELL_d_ values included in Table 5 referring to Poland are higher than in our own study. There are several reasons: first, the findings obtained by the WHO are only estimates, while ours are detailed on the basis of a complete dataset of deaths; second, the values in the table are crude data, while ours are discounted by time and weighted by age; third, the standard life table used for calculation of years of life lost for a death at a given age is based on the projected frontier life expectancy for 2050, with a life expectancy at birth of 92 years. The calculations performed in our present study are based on *Coale and Demeny West Level 26* model life tables.

**Table 5:** Estimated SEYLL_p_ and SEYLL_d_ values for infectious and parasitic diseases in European countries in 2012 by sex.

Country	SEYLL_p_ per 10,000				SEYLL_d_			
	Males	Females	Total	Rank	Males	Females	Total	Rank
Albania	26,65	26,23	26,44	*20*	52,88	46,64	49,60	*39*
Austria	24,45	16,89	20,58	*15*	26,11	17,22	21,45	*16*
Belarus	130,73	42,79	83,63	*38*	48,32	49,86	48,73	*38*
Belgium	38,87	35,99	37,40	*28*	20,13	14,79	17,11	*3*
Bosnia and Herzegovina	27,82	17,39	22,48	*17*	31,11	25,33	28,54	*24*
Bulgaria	59,08	19,78	38,89	*29*	44,54	41,01	43,56	*35*
Croatia	23,66	14,33	18,83	*10*	28,41	21,35	25,14	*20*
Cyprus	23,47	14,54	19,10	*12*	21,46	16,85	19,47	*14*
Czech Republic	33,00	29,80	31,38	*22*	22,51	16,88	19,39	*13*
Denmark	34,14	28,68	31,39	*23*	20,42	15,03	17,53	*6*
Estonia	65,44	22,77	42,55	*32*	42,35	37,97	40,99	*34*
Finland	17,61	14,65	16,11	*7*	21,22	15,77	18,30	*9*
France	42,71	30,92	36,63	*27*	20,59	14,25	17,25	*5*
Germany	43,58	35,19	39,31	*30*	20,19	14,53	17,14	*4*
Greece	24,50	12,05	18,19	*8*	35,52	28,69	32,89	*27*
Hungary	21,55	16,39	18,84	*11*	27,67	19,20	23,03	*19*
Iceland	8,65	10,17	9,40	*1*	20,81	16,42	18,20	*8*
Ireland	20,30	16,46	18,37	*9*	29,40	22,15	25,61	*22*
Italy	41,07	29,27	35,00	*26*	21,94	16,00	18,92	*12*
Latvia	78,37	29,27	51,72	*34*	44,04	32,75	39,82	*33*
Lithuania	88,52	31,28	57,64	*35*	39,09	25,78	33,96	*28*
Luxembourg	33,22	33,79	33,49	*25*	20,44	17,36	18,75	*11*
Malta	15,59	13,35	14,46	*5*	43,66	32,23	37,53	*31*
Montenegro	14,19	7,04	10,57	*3*	34,58	28,62	32,31	*26*
Netherlands	27,82	24,71	26,25	*19*	20,02	16,02	17,90	*7*
Norway	32,48	29,89	31,19	*21*	16,62	12,36	14,27	*1*
Poland	22,56	8,07	15,06	*6*	36,26	30,37	34,41	*29*
Portugal	96,20	42,22	68,36	*36*	29,87	19,28	25,42	*21*
Republic of Moldova	136,59	29,99	80,53	*37*	47,34	48,26	47,51	*37*
Romania	68,75	24,46	46,03	*33*	38,38	34,23	37,15	*30*
Serbia	26,56	13,61	19,94	*13*	33,09	25,39	29,92	*25*
Slovakia	26,26	19,51	22,79	*18*	29,10	23,06	26,10	*23*
Slovenia	12,60	6,68	9,62	*2*	26,14	16,90	21,95	*18*
Spain	41,00	24,52	32,66	*24*	25,92	17,37	21,84	*17*
Sweden	41,95	40,20	41,08	*31*	15,65	13,27	14,39	*2*
Switzerland	23,04	17,38	20,17	*14*	21,11	16,23	18,66	*10*
The former Yugoslav Republic of Macedonia	14,68	9,20	11,94	*4*	39,72	38,20	39,12	*32*
Ukraine	415,09	177,19	286,85	*39*	45,34	46,78	45,81	*36*
United Kingdom	23,45	20,82	22,12	*16*	23,99	18,08	20,75	*15*

Source: [42] and [43]

With regards to standard expected years of life lost due to infectious diseases, calculated in 2012, Poland was placed in 29^th^ position based on the SEYLL_d_ index, and sixth position based on the SEYLL_p_ index [42–43]. It is worth stressing that all the studied values were higher in males than in females, thereby contributing to excess mortality in males and health inequalities between these two sexes. This discrepancy may be associated with the less favourable health behaviours of males as far as prevention and adherence to treatment are concerned, and hence the worse lifestyle of males.

### Limitations of this study and datasets

One of the limitations of the present study was the 14-year duration of the study period, which was not particularly long in the context of evaluation of trends regarding SEYLL. On the other hand, the 14-year study period was long enough to introduce some variation in the quality of reporting and registration of causes of death in Poland. It needs to be emphasized that the data analysed in the records did not include microbiological data, nor the medical histories of the dead persons. The database supplied by the Central Statistical Office is based on data included in the statistical card, which is a part of the death card. The doctor who states the death puts the underlying, secondary and direct causes of death. Since 1 January 1999, each Polish region has been assigned a coder, who assigns the appropriate codes of causes according to the ICD-10 on the basis of a scanned copy of the death cards completed with a written description of the cause of death. The process can be done online, working directly on a server of the Central Statistical Office (CSO) through a work station situated in a statistical office, and offline, by downloading the assigned material from the CSO server to a work station without access to the Internet. The coding doctor contacts the doctor who stated the death by mail or phone in case of doubt. When coding of the cause of deaths, additional procedures safeguarding against possible mistakes are implemented, one being cross-verification of data concerning certain death causes. This procedure is applied to those diseases for which separate registers of incidence are used i.e. infectious diseases, including TB, as well as oncological and mental disorders. The terminology of underlying causes which we use in this article is taken directly from the ICD-10.

## Conclusions

1. Despite a decrease in SDR due to infectious diseases, they remain a serious problem for public health: they contributed to 0.73% of the total number of deaths in Poland in the period 1999 to 2012.
2. Positive trends were observed regarding the SEYLL, SEYLL_p_ and SEYLL_d_ indices calculated for infectious diseases in the study period. However, one person who died of these diseases lost as many as 10.3 years of life.

## Ethics statement

This is a population-based cross-sectional study on the basis of data taken from death cards. Approval was obtained for the study from the Bioethics Committee of the Medical University of Lodz (number RNN/422/12/KB).

## Supporting information

S1 File Database on deaths due to infectious diseases in Poland (1999–2012). (XLSX)
